# Helix-Sense-Selective Polymerization of Phenylacetylenes Having a Porphyrin and a Zinc-Porphyrin Group: One-Handed Helical Arrangement of Porphyrin Pendants

**DOI:** 10.3390/polym11020274

**Published:** 2019-02-06

**Authors:** Masahiro Teraguchi, Nobuyuki Nahata, Takahiro Nishimura, Toshiki Aoki, Takashi Kaneko

**Affiliations:** 1 Department of Chemistry and Chemical Engineering, Niigata University, Ikarashi 2-8050, Nishi-ku, Niigata 950-2181, Japan; tk000211@gmail.com (N.N.); takahiro.nishimura@jpn.dupont.com (T.N.); toshaoki@eng.niigata-u.ac.jp (T.A.); kanetaka@gs.niigata-u.ac.jp (T.K.); 2 Graduate School of Science and Technology, Niigata University, Ikarashi 2-8050, Nishi-ku, Niigata 950-2181, Japan

**Keywords:** helix-sense-selective polymerization, conjugated polymer, poly(phenylacetylenes), one-handed-helical polymer, porphyrin

## Abstract

Newly synthesized two kinds of achiral phenylacetylenes having a free-base- or a zinc-porphyrin (**1** and **Zn1**, respectively) were polymerized by using a chiral rhodium catalyst system, Rh^+^(nbd)[(*η*^6^-C_6_H_5_)B^–^(C_6_H_5_)_3_] catalyst and (*R*)-(+)- or (*S*)-(–)-1-phenylethylamine ((*R*)- or (*S*)-PEA, respectively) cocatalyst. Poly(**1**) and poly(**Zn1**) in THF showed a Cotton signal at the absorption region of the porphyrin and the main chain in the circular dichroism (CD) spectra. This result suggests that poly(**1**) and poly(**Zn1**) exist in a conformation with an excess of one-handed helix sense and the porphyrin moiety arranged in chiral helical fashion. The one-handed helical structure of poly(**1**) could be sustained in a mixture of THF/HMPA (10/2, *v*/*v*) due to stabilizing by stacking effect of porphyrin moieties along the main chain. This is the first example about helix-sense-selective polymerization by using Rh^+^(nbd)[(*η*^6^-C_6_H_5_)B^–^(C_6_H_5_)_3_] catalyst. Additionally, poly(**Zn1**) showed about 10 times larger CD intensity in comparison with poly(**1**). This result suggests the regularity of arrangement of the porphyrin in poly(**Zn1**) is higher compared with poly(**1**). Spatial arrangement of porphyrins was achieved by utilizing a one-handed helical poly(phenylacetylenes) as a template.

## 1. Introduction

The synthesis of one-handed helical polyacetylenes has attracted much attention due to their interesting applications such as chiral sensors, chiral catalysts, optical resolution, microelectronic devices, organic magnetic materials, and so on [1,2,3,4,5,6]. The one-handed helical polymers are one of the stereoregular polymers and are also useful as a template for spatially arranging functional groups introduced in the side chain to create novel functional materials.

Porphyrin, which is a widely conjugated, large cyclic, and planar molecule, is attracting the attention of many researchers in various fields because of its functions such as catalysis, molecular recognition, fluorescence, energy transfer, and light-harvesting [7,8,9,10]. Porphyrin is also expected to play an important role in realizing artificial photosynthesis [11,12]. Particularly, to realize efficient light-harvesting systems, precise spatial arrangement of porphyrins is considered to be the key. A number of studies on the synthesis, fluorescence, and electronic properties of polymer with porphyrin moieties as pendants have been reported [13,14,15,16], but there are few reports on polyacetylene [17]. The challenge of the arrangement of functional molecules, such as porphyrins, remain of interest, utilizing a rigid stereoregular polymer such as a one-handed helical polyacetylene as a template.

The synthetic methods of one-handed helical polyacetylenes are roughly classified into two categories, i.e., asymmetric induced polymerization (AIP) and helix-sense-selective polymerization (HSSP). AIP, in which chiral substituted acetylenes are used as a monomer, is a simple method to obtain one-handed helical polyacetylenes [18,19,20,21,22,23,24]. However, the poly(substituted acetylenes) synthesized by AIP have two chiral sources, i.e., chiral side groups and one-handed helical main chain to causes which complicates the understanding of their chiral functions. HSSP is another method used to obtain one-handed helical polyacetylenes in which achiral acetylene monomers and other chiral sources such as initiators [25,26,27,28], additives [29,30,31], and a polymerization field [32,33,34] are used. The obtained one-handed helical substituted polyacetylenes having no other chiral moieties in the side or end groups.

Previously, we have reported the HSSP of 4-dodecyloxy-3,5-bis(hydroxymethyl)phenylacetylene (DoDHPA) by a chiral catalyst system, [Rh(nbd)Cl]_2_–(*R*)-(+)-1-phenylethylamine ((*R*)-PEA) [25]. The resulting polymer showed large Cotton effects in the CD spectrum attributed to one-handed helical conformation in a solution which is kinetically stabilized by intramolecular hydrogen bonds. Furthermore, HSSP is more valuable than AIP because it could synthesize right-handed or left-handed helical polyacetylenes by using a catalytic amount of chiral substances.

**Scheme 1:**
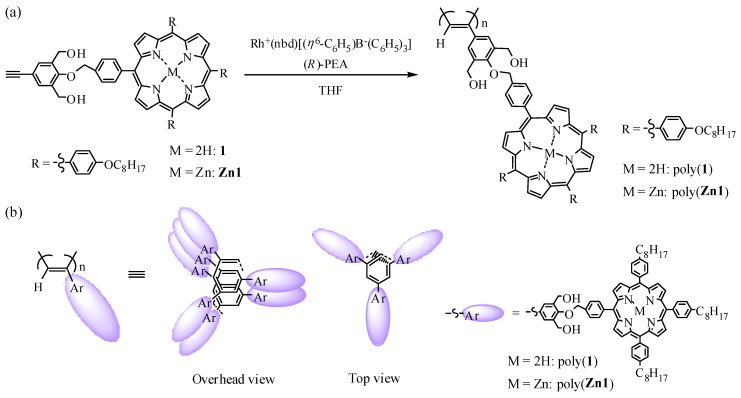
(**a**) Helix-sense-selective polymerization (HSSP) of monomers having porphyrin moiety; (**b**) schematic structures of poly(**1**) and poly(**Zn1**) synthesized by HSSP (the overhead view and the top view are represented using 7 and 3 repeating units, respectively, for simplicity).

In this paper, we report the synthesis and the helix-sense-selective polymerization of a phenylacetylene having two hydroxylmethyl groups and a porphyrin moiety by a new chiral rhodium catalyst system (Scheme 1). The spatial arrangement of porphyrins was achieved by utilizing a one-handed helical poly(phenylacetylenes) as a template.

## 2. Experimental Section

### 2.1. Materials

A Rh^+^(nbd)[*η*^6^-C_6_H_5_)B^–^(C_6_H_5_)_3_] (nbd = 2,5-norbornadiene) catalyst was synthesized according to the method reported by Schrock et al. [35] (*S*)-(−)-or (*R*)-(+)-1-Phenylethylamine was purchased from Tokyo Chemical Industry Co. (Tokyo, Japan) and used without further purification. Triethylamine, CHCl_3_ and CH_2_Cl_2_ were distilled from CaH_2_ before use. 4-Hydroxy-3,5-bis(hydroxymethyl)phenylacetylene was synthesized according to our previous report [36].

### 2.2. Monomer Synthesis

#### 2.2.1. Synthesis of 4-(4-((6,11,16-tri(4-*n*-octyloxyphenyl)porphynyl)benzyloxy)-3,5-bis(hydroxymethyl)phenylacetylene(**1**)

**Scheme 2:**
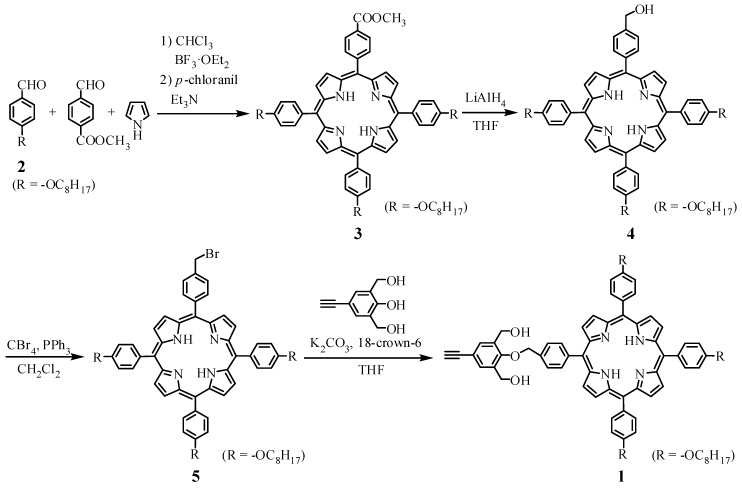
Synthetic route of **1**.

The synthetic route of **1** is shown in Scheme 2.

##### 4-*n*-Octyloxybenzaldehyde(**2**)

4-hydroxybenzaldehyde (20.0 g, 164 mmol), potassium carbonate (45.3 g, 328 mmol), and potassium iodide (27.2 g, 164 mmol) were taken in acetone (275 mL) and stirred at reflux temperature under a nitrogen atmosphere for 2 h. 1-Bromooctane (31.4 mL, 180 mmol) was added dropwise to the above hot reaction mixture. The solution was refluxed for additional 24 h under a nitrogen atmosphere. After the reaction, the solution was cooled to room temperature. Then the volatile solvent, acetone, was removed under a reduced pressure, and the reaction residue was poured into a large amount of water (550 mL). The solution was extracted with dichloromethane, and the organic layer was washed with 2% NaOH aq. (825 mL) and brine. Then the organic solution was dried over anhydrous Na_2_SO_4_ and concentrated to give a pale yellow oil as a crude product. It was further purified by silica gel column chromatography (hexane:ethyl acetate = 9:1) to give the desired product as a yellow viscous liquid. R_f_ = 0.33; Yield = 33.5 g (87.3%).

^1^H NMR (CDCl_3_, 400 MHz), δ: 9.85 (s, 1H, Ar–CHO), 7.80 (d, 2H, Ar–H), 6.97 (d, 2H, Ar–H), 4.01 (t, 2H, Ar–OCH_2_), 1.82–1.76 (quit, 2H, Ar–OCH_2_CH_2_CH_2_), 1.48–1.42 (quint, 2H, Ar–OCH_2_CH_2_CH_2_CH_2_), 1.37–1.22 (8H, aliphatic), 0.88 (t, 3H, –CH_3_).

##### 5-(4-Methoxycarbonylphenyl)-10,15,20-tri(4-*n*-octyloxyphenyl)porphyrin(**3**)

A mixture of pyrrole (0.56 mL, 8.0 mmol), methyl terephthalaldehydate (328 mg, 2.0 mmol), and **2** (1.43 mL, 6.0 mmol) in chloroform (800 mL) was stirred at room temperature under a nitrogen atmosphere for 15 min. BF_3_·Et_2_O (0.1 mL) was added in one portion, and the reaction mixture was stirring at room temperature for an additional 30 min. Triethylamine (0.2 mL, 1.43 mmol), and chloranil (2.2 g, 9.0 mmol) were added, and the reaction mixture was shaded from the ambient light and further stirred at reflux temperature for 1 h. It was cooled, chloroform was removed under vacuum, and the residue was purified by silica gel column chromatography (hexane:dichloromethane = 1:2) to give the desired product as a purple solid. R_f_ = 0.50; Yield = 0.200 g (9.34%).

^1^H NMR (CDCl_3_, 400 MHz), δ: 8.97–8.68 (m, 8H, β–H), 8.44(d, 2H, Ar–H), 8.30 (d, 2H, Ar–H), 8.16–8.05 (m, 6H, Ar–H), 7.33–7.22 (m, 6H, Ar–H), 4.25 (t, 6H, –OC_8_H_17_), 4.11 (s, 3H, COOCH_3_), 1.99 (tt, 6H, –OC_8_H_17_), 1.70–1.56 (m, 30H, –OC_8_H_17_), 0.94 (t, 9H, –OC_8_H_17_).

##### 5-(4-Bromomethylphenyl)-10,15,20-tri(4-*n*-octyloxyphenyl)porphyrin(**5**)

Compound **3** (366 mg, 0.346 mmol) was dissolved in dry THF (3.46 mL). Lithium aluminum hydride (40.0 mg, 1.04 mmol) was added slowly to the reaction mixture at 0 °C. The reaction mixture was stirred at room temperature under a nitrogen atmosphere for 30 min. The reaction mixture was poured into 2N HCl (15 mL) at 0 °C. THF was removed under vacuum, and the residue was washed with brine. The organic layer was dried over anhydrous Na_2_SO_4_ and condensed to get a green solid crude product. The crude product was taken to the next step without further purification. The crude **4** (267 mg, 0.259 mmol), carbon tetrabromide (292 mg, 0.880 mmol), and triphenylphosphin in dichloromethane (3.63 mL) was stirred at room temperature under a nitrogen atmosphere for 60 min. The reaction mixture was washed with saturated NaHCO_3_ aqueous and brine. The organic layer was dried over anhydrous Na_2_SO_4_ and condensed to achieve a purple solid product. It was further purified by silica gel column chromatography (hexane:dichloromethane = 2:3) to give the desired product as purple solid. R_f_ = 0.47; Yield = 85.5 mg (30.2%).

^1^H NMR (CDCl_3_, 400 MHz), δ: 8.96–8.77 (m, 8H, β–H), 8.51(d, 2H, Ar–H), 8.27 (d, 2H, Ar–H), 8.15–8.08 (d, 6H, Ar–H), 7.32–7.21 (m, 6H, Ar–H), 4.97 (s, 2H, –CH_2_Br), 4.26 (t, 6H, –OC_8_H_17_), 1.99 (tt, 6H, –OC_8_H_17_), 1.66–1.22 (m, 30H, –OC_8_H_17_), 0.94 (t, 9H, –OC_8_H_17_).

##### 4-(4-((6,11,16-Tri(4-*n*-octyloxyphenyl)porphynyl)benzyloxy)-3,5-bis(hydroxymethyl)phenylacetylene(**1**)

Compound **5** (210 mg, 0.183 mmol), potassium carbonate (37.9 mg, 0.274 mmol), 18-crown-6 (72.3 mg, 0.274 mmol), and 4-hydroxy-3,5-bis(hydroxymethyl)phenylacetylene^36^ (48.7 mg, 0.274 mmol) in dry THF (1.83 mL) was stirred at 50 °C under a nitrogen atmosphere for 24 h. It was then cooled, the THF was removed under vacuum, and the residue was washed with brine. The organic layer was dried over anhydrous Na_2_SO_4_ and condensed to get a purple solid as product. It was further purified by silica gel column chromatography (dichloromethane:ethyl acetate = 20:1) to give the desired product as purple solid. R_f_ = 0.23; Yield = 80.0 mg (36.7%).

^1^H NMR (CDCl_3_, 400 MHz), δ: 8.95–8.74 (m, 8H, β–H), 8.26 (d, 2H, Ar–H), 8.15–8.05 (m, 6H, Ar–H), 7.80 (d, 2H, Ar–H), 7.63 (s, 2H, Ar–H), 7.32–7.23 (m, 6H, Ar–H), 5.31 (s, 2H, –O–CH_2_–), 4.88 (d, 4H, –CH_2_OH), 4.25 (t, 6H, –OC_8_H_17_), 3.10 (s, 1H, C≡C–H), 1.98 (tt, 6H, –OC_8_H_17_), 1.69–1.31 (m, 30H, –OC_8_H_17_), 0.92 (t, 9H, –OC_8_H_17_).

#### 2.2.2. Synthesis of 4-(4-((6,11,16-tri(4-*n*-octyloxyphenyl)zincporphynyl)benzyloxy)-3,5-bis(hydroxymethyl)phenylacetylene (**Zn1**)

**Scheme 3:**
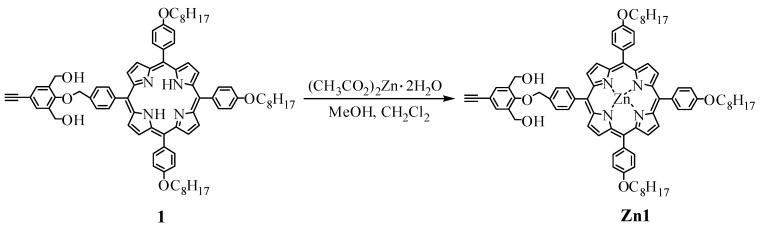
Synthetic route of **Zn1**.

The synthetic route of **Zn1** is shown in Scheme 3.

Compound **1** (18.3 mg, 0.0228 mmol) was dissolved in dichloromethane (2.60 mL) and methanol (0.35 mL). Zinc acetate dihydrate (28.4 mg, 0.129 mmol) was added slowly to the reaction mixture. The reaction mixture was stirred at room temperature under a nitrogen atmosphere for 3 h. The reaction mixture was washed with brine. The organic layer was dried over anhydrous Na_2_SO_4_ and condensed to get a purple solid preproduct. This was further purified by silica gel column chromatography (dichloromethane:ethyl acetate = 10:1) to give the desired product as purple solid. R_f_ = 0.50; Yield = 15.0 mg (77.8%).

^1^H NMR (CDCl_3_, 400 MHz)δ: 8.94–8.80 (m, 8H, β–H), 8.27 (d, 2H, Ar–H), 8.15–8.08 (m, 6H, Ar–H), 7.83 (d, 2H, Ar–H), 7.65 (s, 2H, Ar–H), 7.32–7.25 (m, 6H, Ar–H), 5.09 (s, 2H, –O–CH_2_–), 4.92 (d, 4H, –CH_2_OH), 4.26 (t, 6H, –OC_8_H_17_), 3.11 (s, 1H, C≡C–H), 2.00 (tt, 6H, –OC_8_H_17_), 1.70–1.32 (m, 30H, –OC_8_H_17_), 0.95 (t, 9H, –OC_8_H_17_).

### 2.3. Polymerization

A solution of Rh^+^(nbd)[*η*^6^-C_6_H_5_)B^–^(C_6_H_5_)_3_] (2.30 mg, 4.47 μmol) and (*S*)- or (*R*)-phenylethylamine (PEA) (5.70 μL, 44.7 μmol) in THF (0.218 mL) was added to a solution of **1** (23.3 mg, 19.3 μmol) in THF (0.174 mL). The reaction solution was stirred at room temperature for 24 h. The crude polymer was purified by reprecipitation of the THF solution into a large amount of methanol and dried in vacuo to give a purple solid. **Zn1** was polymerized as the same procedure of **1**. The yields, average molecular weights, polydispersity, and color of the polymers are summarized in Table 1.

### 2.4. Measurements

^1^H NMR spectra (400 MHz) were recorded on a FT-NMR 400MR spectrometer (Varian, Palo Alto, CA, USA). Average molecular weights (*M*_n_ and *M*_w_) and polydispersity ratio were determined by gel-permeation chromatography on two Shodex columns (KF-807Lx2, eluent THF) in a liquid chromatograph device (JASCO, Tokyo, Japan) equipped with UV detector (UV-2070) and calibrated using polystyrene standards. CD/UV-vis spectra were recorded on a J-720WI spectropolarimeter (JASCO, Tokyo, Japan) with a Peltier temperature control. Fluorescence emission spectra were recorded on a FP-6500 spectrophotometer (JASCO, Tokyo, Japan).

## 3. Results and Discussions

Two kinds of monomers, **1** and **Zn1** were synthesized with reference to the literature methods (Scheme 2 and Scheme 3) [37,38,39]. No polymer was obtained when [Rh(nbd)Cl]_2_–(*R*)-PEA and [Rh(nbd)Cl]_2_–Et_3_N catalysts, which were highly active for the polymerization of a wide range of phenylacetylene derivatives, were applied for the polymerization of **1** at first. The monomer was recovered quantitatively.

On the other hand, zwitterionic Rh complex, Rh^+^(nbd)[*(η*^6^-C_6_H_5_)B^–^(C_6_H_5_)_3_] was effective to polymerize **1** and **Zn1** to give corresponding polymers in good yields in the presence of (*R*)- or (*S*)-PEA as a chiral cocatalyst, as shown in Table 1. It is assumed that the porphyrin moiety of monomer **1** deactivates the [Rh(nbd)Cl]_2_–amine catalyst because of its high coordination ability. In contrast, it is considered that the zwitterionic Rh catalyst could polymerize **1** because the bulky tetraphenylborate ligand could inhibit the approach of porphyrin to Rh center.

Poly(**1**) and poly(**Zn1**) were purple and dark purple solids respectively with relatively high molecular weights (weight-average molecular weights (*M*_w_) 4 × 10^4^ and 25.7 × 10^4^, respectively). The *M*_w_ of poly(**Zn1**) was about 6 times higher than that of poly(**1**). This result seems to be due to a more rigid polymer structure of poly(**Zn1**) than that of poly(**1**). It seems that zinc-porphyrins in poly(**Zn1**) interact more strongly between intramolecular side groups than free-base type porphyrins in poly(**1**). This idea is also supported by the CD measurements and fluorescence measurements in the following part of this paper.

Both polymers were soluble in toluene, chloroform, and THF and insoluble in hexane, diethyl ether, methanol, and acetone.

**Figure 1:**
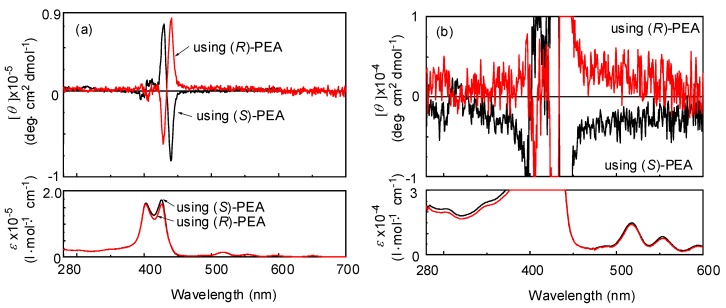
(**a**) CD and UV-vis spectra of poly(**1**) in THF at 20 °C; (**b**) enlargement of the CD and UV-vis spectra in (a) for Y-axis.

The circular dichroism (CD) spectra of the poly(**1**) obtained using (*R*)-PEA and (*S*)-PEA as cocatalyst are shown in Figure 1. The large positive and negative Cotton effects around 420 nm derived from porphyrin moieties were observed for the polymers and both spectra were complete mirror images each other (Figure 1a). In the UV-vis absorption spectra (Figure 1a, bottom), the polymers showed a split Soret band due to porphyrin groups around 420 nm, indicating a highly regular face-to-face arrangement of the porphyrin pendants [40]. These results indicate the porphyrin groups were arranged in a chiral helical fashion along the polymer backbone. Poly(**1**) obtained using (*R*)-PEA and (*S*)-PEA showed a positive and a negative exciton split CD signal at the Soret band, respectively. Poly (**1**) synthesized using (*R*)-PEA showed the positive CD couplet, a cotton signal with positive-to-negative pattern, ongoing from longer to short wavelength. Using the exciton chirality method [41,42,43], spatial arrangement of porphyrin groups in the poly(**1**) obtained using (*R*)-PEA was proven to be regularly arranged in right-handed helical sense. By using (*R*)-PEA or (*S*)-PEA, we could successfully adjust the helix sense of porphyrin arrangement to right handed or left handed.

**Figure 2:**
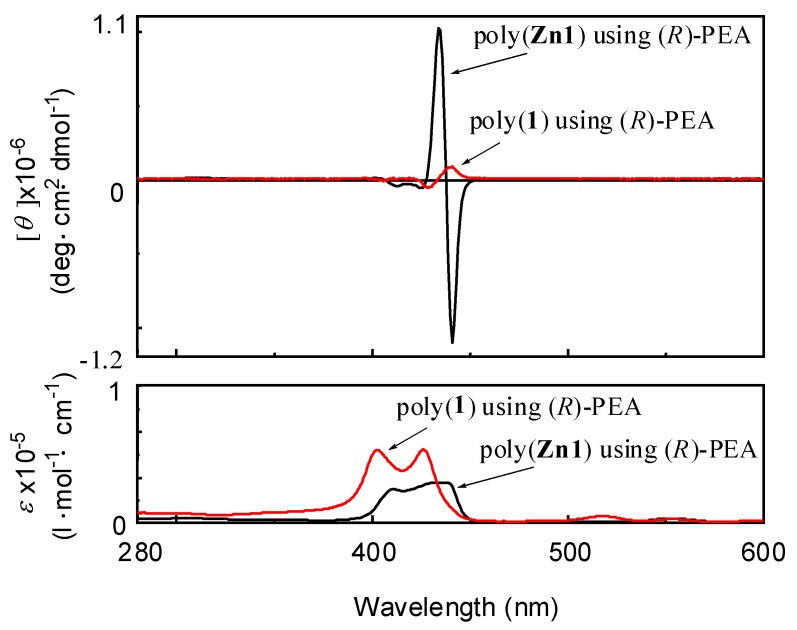
CD and UV-vis spectra of poly(**1**) and poly(**Zn1**).

In Figure 2, CD spectra of the poly(**Zn1**) using (*R*)-PEA compared with poly(**1**) are shown. The CD intensity of poly(**Zn1**) was about 10 times larger than that of poly(**1**), which suggests that the regularity of the porphyrin arrangement of poly(**Zn1**) is much higher than that of poly(**1**).

Furthermore, the broad Cotton signals due to the one-handed helical backbone of polyacetylenes in the range of 350–500 nm were confirmed in the enlargement of the CD spectra (Figure 1(b)). Judging from these results, HSSP of **1** was achieved by using Rh^+^(nbd)[(*η*^6^-C_6_H_5_)B^–^(C_6_H_5_)_3_]–(*R*)- or (*S*)-PEA. To the best of our knowledge, this is the first report about HSSP using a Rh^+^(nbd)[(*η*^6^-C_6_H_5_)B^–^(C_6_H_5_)_3_] catalyst.

**Figure 3:**
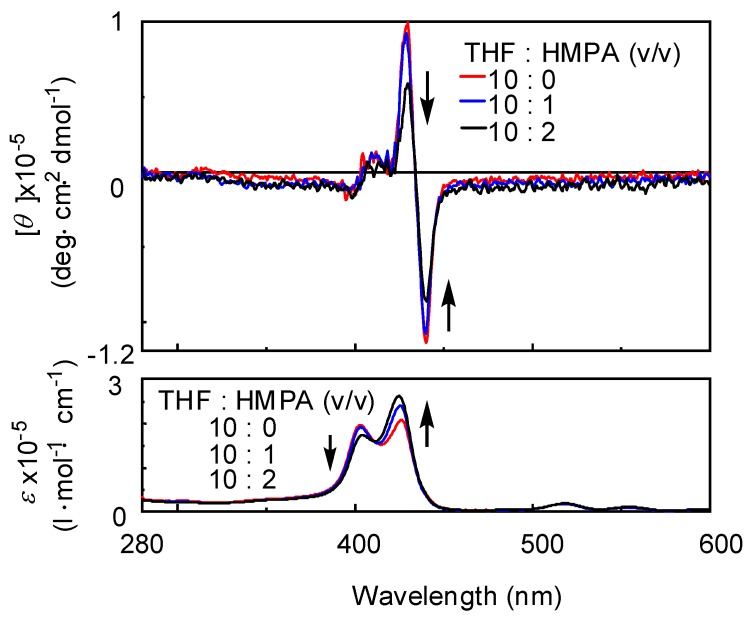
CD and UV-vis spectra of poly(**1**) measured in THF/HMPA at room temperature.

To investigate the stability of the helical structure stabilized with intramolecular hydrogen bonds, the CD spectra of poly(**1**) in mixed solvents of THF/HMPA with various ratios were measured (Figure 3). When the polar solvent, HMPA, was added until 10/1 and 10/2 of the volume ratio of THF/HMPA, the intensity of the Cotton signal weakened to 91% and 64% of that of the original respectively. This suggests that the helical structure was partially deformed by the polar solvent cleaving the intramolecular hydrogen bond. This idea was verified by IR measurements in THF/HMPA solution (Figure S1, Supplementary Materials). In the case of one-handed helical poly(DoDHPA) [25] reported previously, which has a dodecyloxy groups at para position instead of the porphyrin groups, the Cotton signals completely disappeared in 10:1 (*v*/*v*) of THF/HMPA (Figure S2, Supplementary Materials). In other words, the one-handed helical structure of poly(**1**) was more stable than that of poly(DoDHPA). Therefore, it seems that the one-handed helical structure of poly(**1**) is stabilized by the stacking of porphyrin along the main chain.

**Figure 4:**
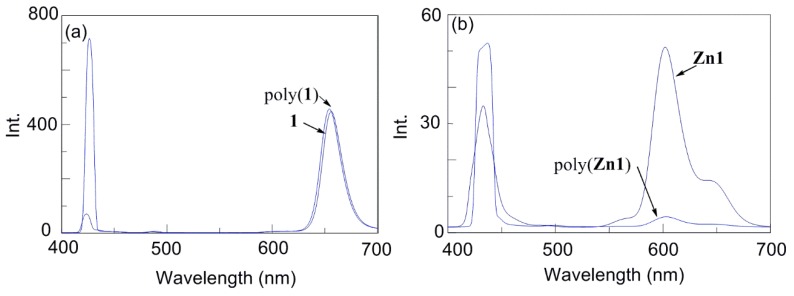
Fluorecence spectra of (**a**) **1** and poly(**1**), excited at 420 nm; (**b**) **Zn1** and poly(**Zn1**), excited at 425 nm (**Zn1**), excited at 430 nm (poly(**Zn1**)); in THF at 20 °C, cell length = 10 mm, *c* = 0.5 × 10^−6^ M based on porphyrin unit).

**Figure 5:**
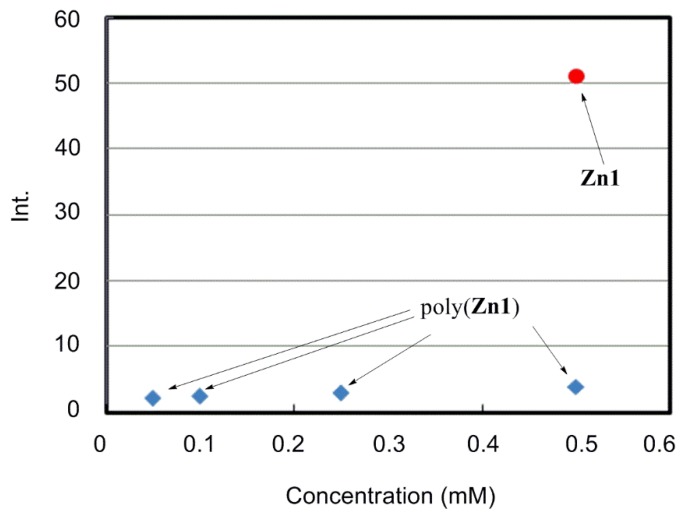
Fluorecence intensity of **Zn1** and poly(**Zn1**) in THF at 20 °C.

The fluorescence spectra of poly(**1**) and poly(**Zn1**) together with that of each monomer are shown in Figure 4. The UV-vis spectra of **1**, poly(**1**), **Zn1**, and poly(**Zn1**) are available in Supplementary Materials (Figures S3 and S4). The fluorescence spectra of both **1** and poly(**1**) with excitation at 420 nm showed a similar fluorescence band at 655 nm. On the other hand, **Zn1** showed a fluorescence band at 600 nm, whereas strong quenching was observed in the fluorescence spectra of poly(**Zn1**). The concentration dependence of the fluorescence quenching of poly(**Zn1**) was also investigated (Figure 5). The fluorescence quenching of poly(**Zn1**) showed no concentration dependence, and fluorescence intensity was almost same in the range from 0.5 to 0.05 μM. Therefore it is assumed that porphyrin moieties of poly(**Zn1**) strongly interact between side groups in a polymer chain.

## 4. Conclusions

Helix-sense-selective polymerization of two kinds of achiral phenylacetylenes having a free-base- or a zinc-porphyrin (**1** and **Zn1**, respectively) and two hydroxymethyl groups was achieved by using a chiral rhodium catalyst system, Rh^+^(nbd)[(*η*^6^-C_6_H_5_)B^–^(C_6_H_5_)_3_] catalyst and (*R*)-(+)- or (*S*)-(−)-PEA, cocatalyst. This is the first example of HSSP of acetylenes using a Rh^+^(nbd)[(*η*^6^-C_6_H_5_)B^–^(C_6_H_5_)_3_] catalyst. Poly(**Zn1**) showed a CD intensity approximately ten times larger in comparison with poly(**1**). This result suggests the regularity of arrangement of the porphyrin in poly(**Zn1**) is higher compared with poly(**1**). The spatial arrangement of porphyrins was achieved by utilizing a one-handed helical poly(phenylacetylenes) as a template.

## Figures and Tables

**Table 1:** Polymerization results of phenylacetylene having porphyrin moiety ^a^.

Run	Monomer	Cocat.	Yield (%)	*M*_w_^b^ (×10^4^)	*M*_w_/*M*_n_	Color
1	**1**	(*R*)-PEA	89.5	4.16	1.42	purple
2	**1**	(*S*)-PEA	87.8	4.35	1.33	purple
3	**Zn1**	(*R*)-PEA	90.3	25.7	1.42	dark purple

^a^ Polymerized in THF for 24 h at r.t. using Rh^+^(nbd)[B^–^(C_6_H_5_)_4_] as catalyst; [M] = 0.1 M, [M]/[cat.] = 50, [cocat.]/[cat.] = 10. ^b^ Determined by GPC (THF, PSt standard).

## Supplementary Materials

The following are available online at https://www.mdpi.com/2073-4360/11/2/274/s1, Figure S1, IR spectra of poly(**1**) in THF/HMPA at room temperature. Figure S2, CD and UV-vis spectra of poly(DoDHPA) in THF/HMPA at room temperature. Figure S3, UV-vis spectra of **1** and poly(**1**) in THF at 20 °C (cell length = 10 mm)(*c* = 0.0005 mM). Figure S4, UV-vis spectra of **Zn1** and poly(**Zn1**) in THF at 20 °C (cell length = 10 mm) (*c* = 0.0005 mM). ^1^H NMR spectra of monomer and intermediates, and mass spectrum of **1**.
