# Supplementary material for: Helix-Sense-Selective Polymerization of Phenylacetylenes Having a Porphyrin and a Zinc-Porphyrin Group: One-Handed Helical Arrangement of Porphyrin Pendants
Source: Polymers (Basel). 2019 Feb 6;11(2):274. doi: 10.3390/polym11020274 (PMC6419054; doi:10.3390/polym11020274)
Supplement: Supplementary file 1 [file polymers-11-00274-s001.pdf]

Supporting Information

Helix-sense-selective Polymerization of Phenylacetylenes Having a Porphyrin and a Zinc-Porphyrin Group: One-handed Helical Arrangement of Porphyrin Pendants

Masahiro Teraguchi,*[1,2] Nobuyuki Nahata,[1] Takahiro Nishimura,[1] Toshiki Aoki,[1,2] Takashi Kaneko[1,2]

[1] Department of Chemistry and Chemical Engineering, Niigata University, Ikarashi 2-8050,Nishi-ku, Niigata 950-2181

[2] Graduate School of Science and Technology, Niigata University, Ikarashi 2-8050,Nishi-ku, Niigata 950-2181

* Corresponding author. Tel./Fax: +81-25-262-7491. E-mail address: teraguti@eng.niigata-u.ac.jp (M. Teraguchi).

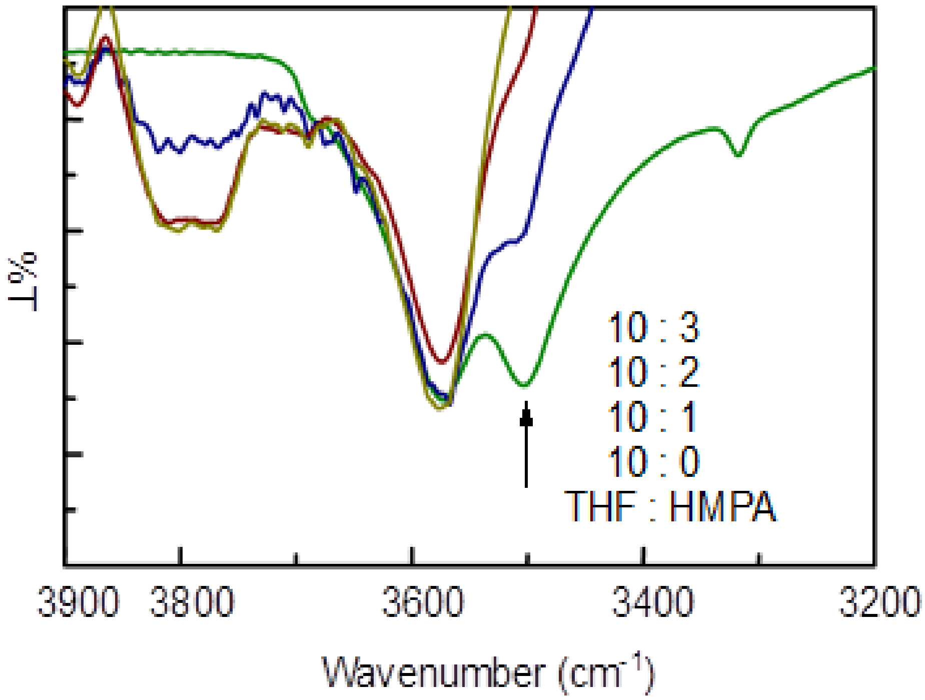

Figure S1. IR spectra of poly(**1**) in THF/HMPA at room temperature.

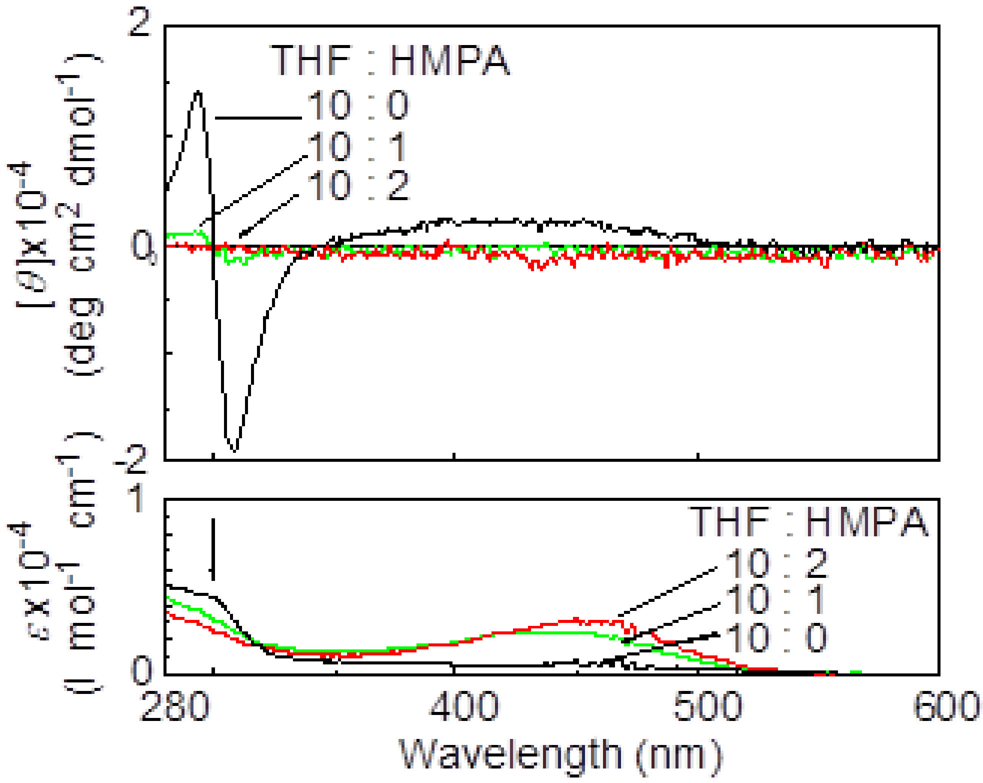

Figure S2. CD and UV-vis spectra of poly(**DoDHPA**) in THF/HMPA at room temperature.

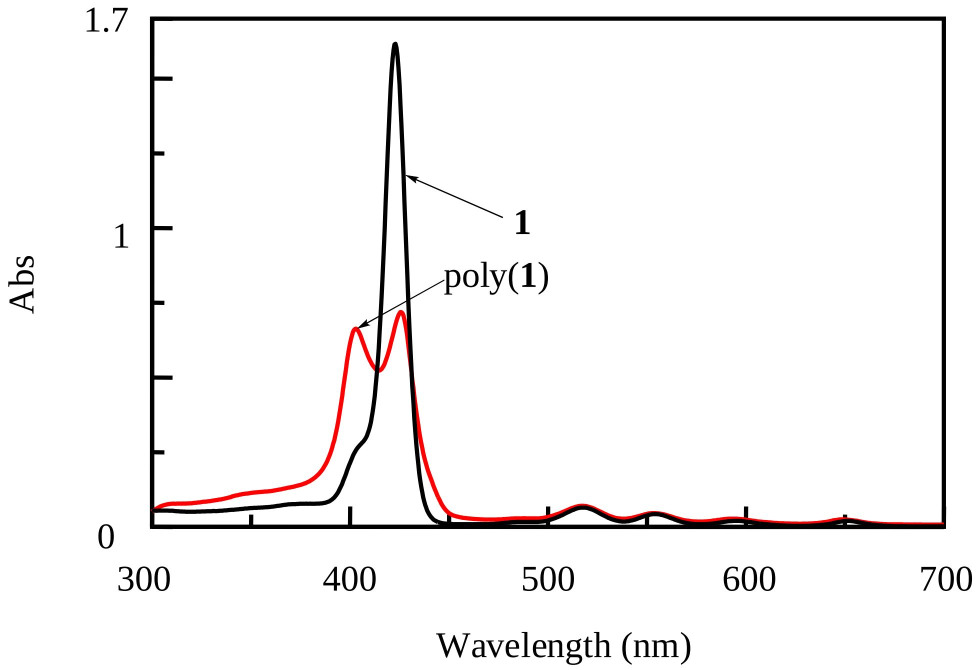

Figure S3. UV-vis spectra of **1** and poly(**1**) in THF at 20°C (cell length = 10 mm)(M = 0.0005 mM).

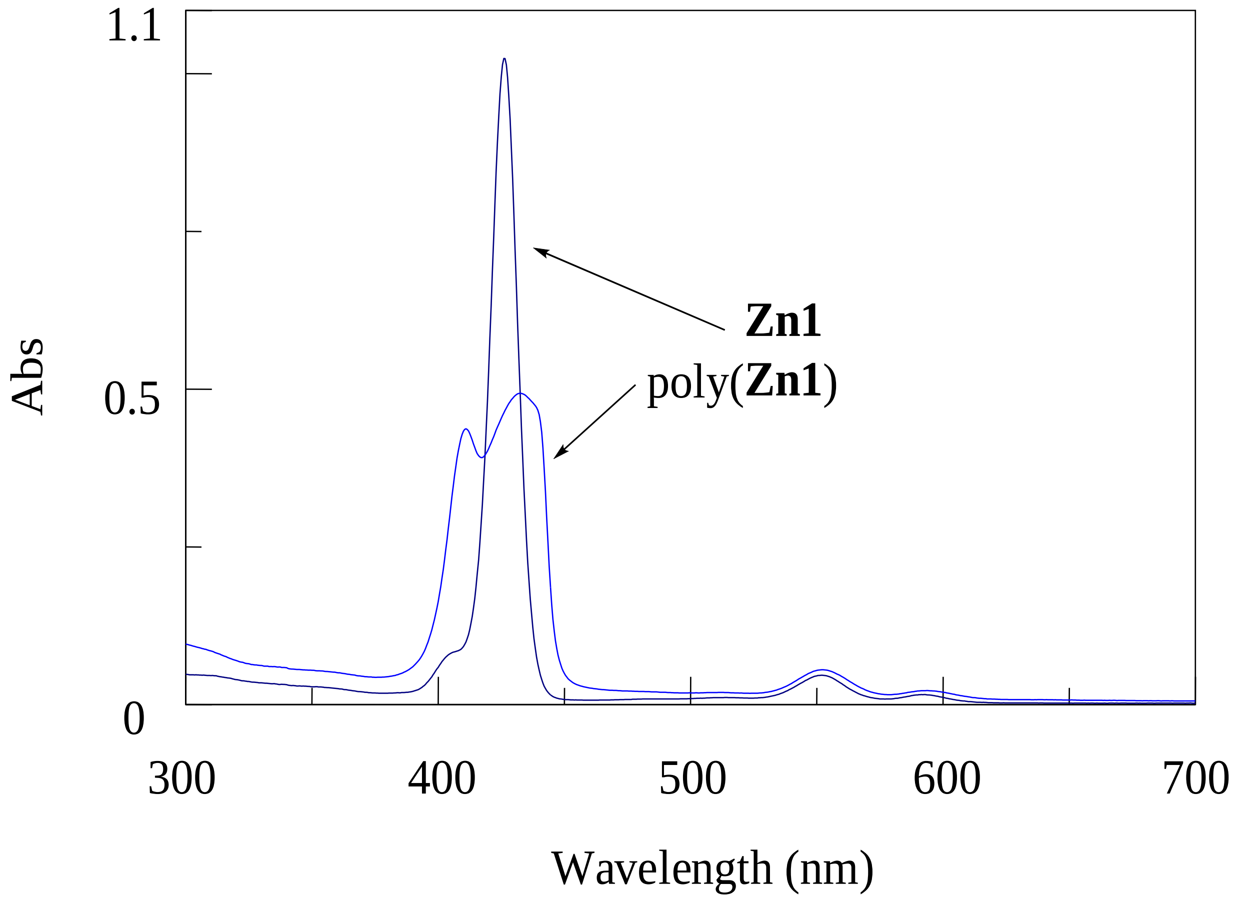

Figure S4. UV-vis spectra of **Zn1** and poly(**Zn1**) in THF at 20 ^{o}C (cell length = 10 mm) (M = 0.0005 mM).

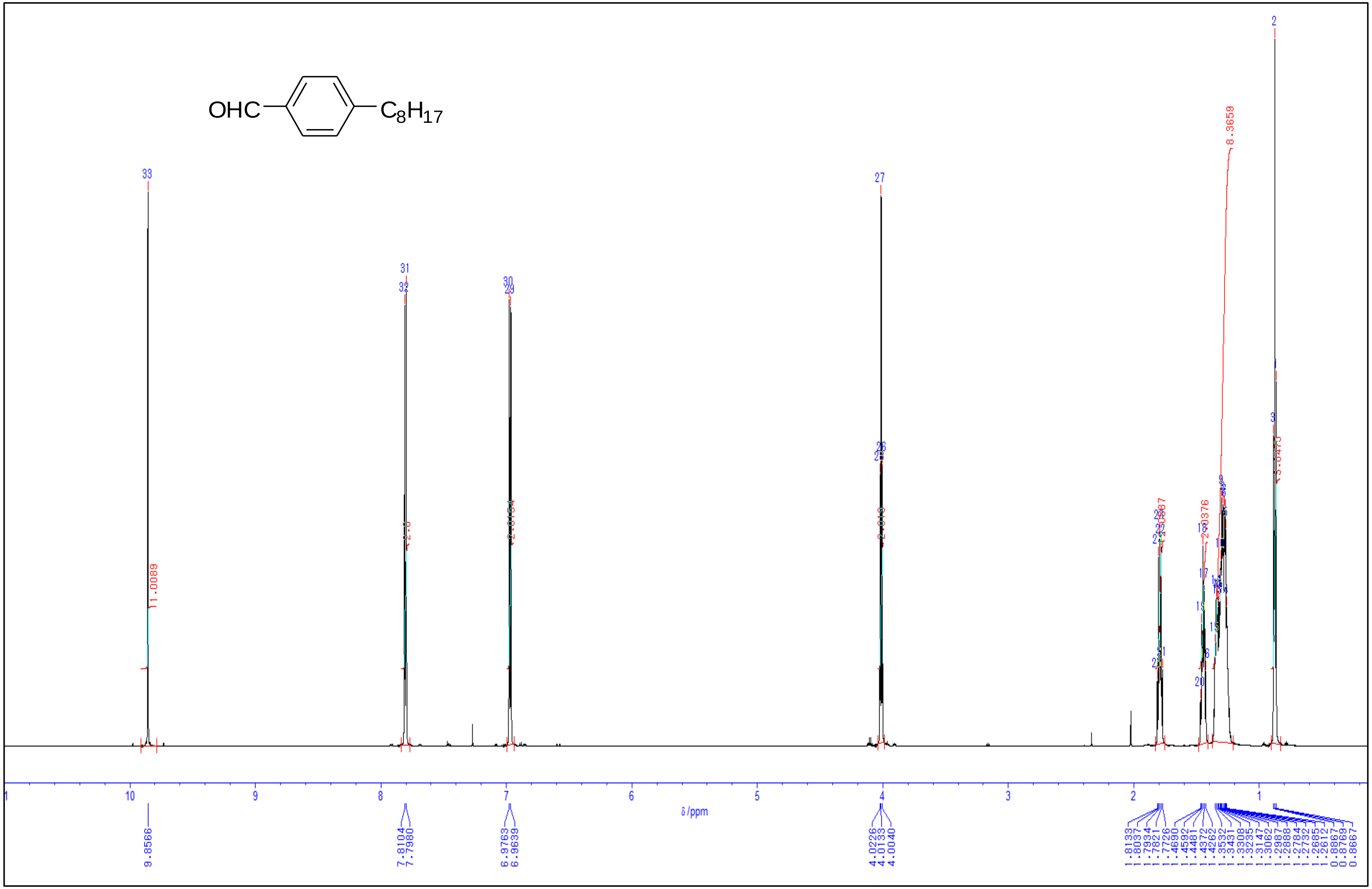

OHC
C8H17
δ/ppm

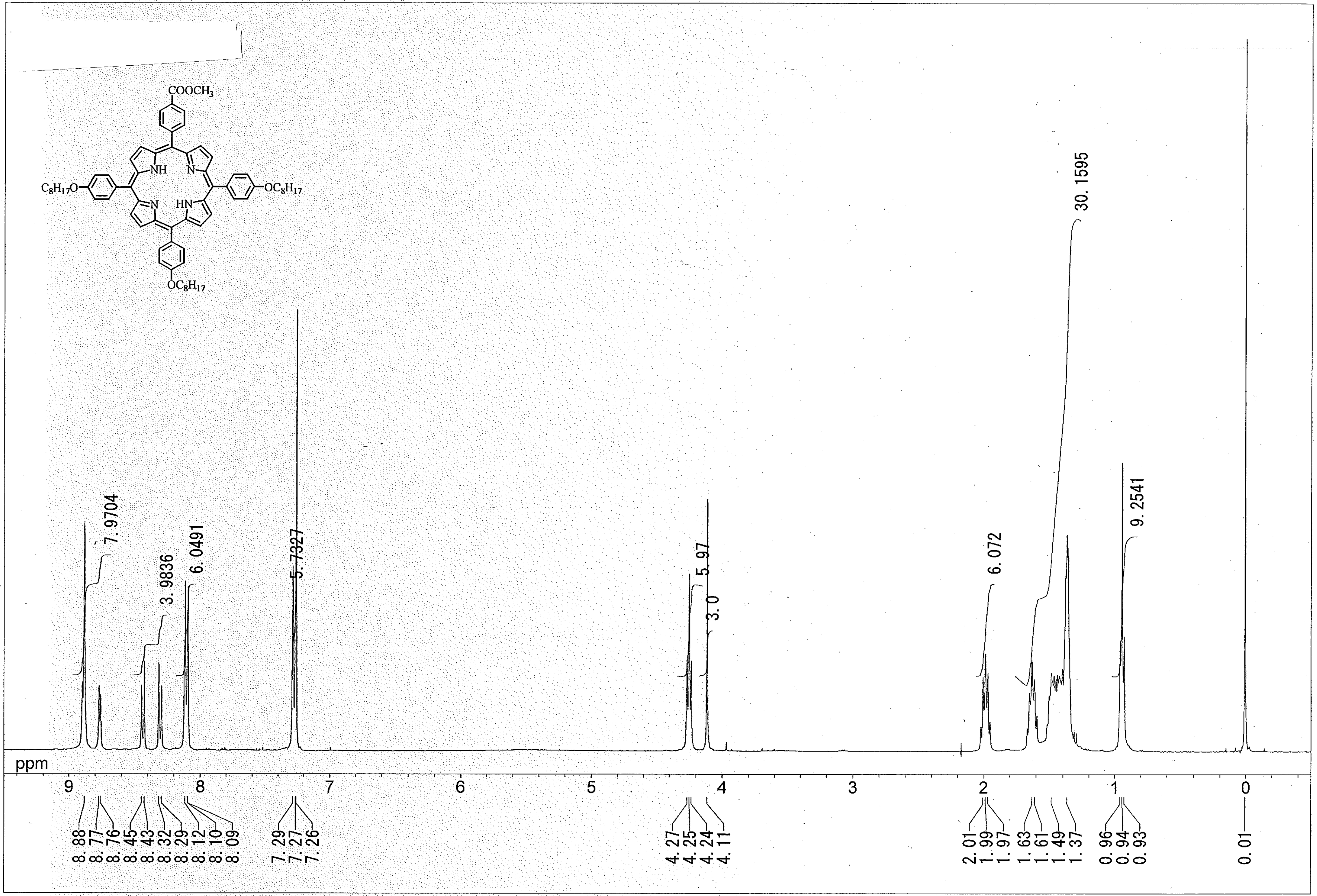
COOCH3
NH
N
HN
C8H17O
OC8H17
OC8H17
7.9704
3.9836
6.0491
5.7327
5.97
3.0
6.072
30.1595
9.2541
ppm
9
8
7
6
5
4
3
2
1
0
8.88 8.77 8.76 8.45 8.43 8.32 8.29 8.12 8.10 8.09
7.29 7.27 7.26
4.27 4.25 4.24 4.11
2.01 1.99 1.97 1.63 1.61 1.49 1.37
0.96 0.94 0.93
0.01

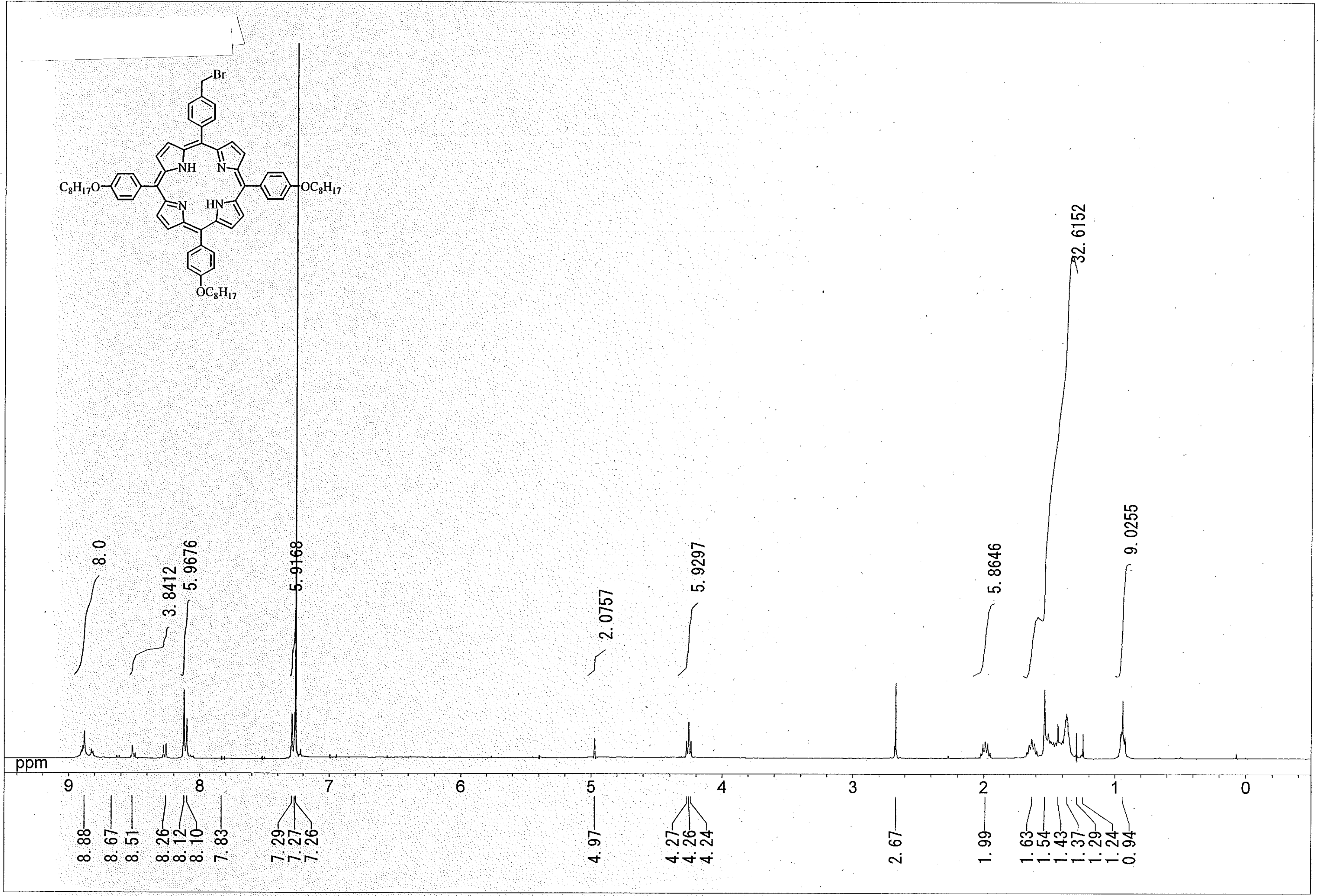
Br
NH
N
N
HN
$C_8H_{17}O$
OC_8H_{17}
OC_8H_{17}
ppm
9
8
7
6
5
4
3
2
1
0
8.0
3.8412
5.9676
5.9168
2.0757
5.9297
5.8646
32.6152
9.0255
8.88
8.67
8.51
8.26
8.12
8.10
7.83
7.29
7.27
7.26
4.97
4.27
4.26
4.24
2.67
1.99
1.63
1.54
1.43
1.37
1.29
1.24
0.94

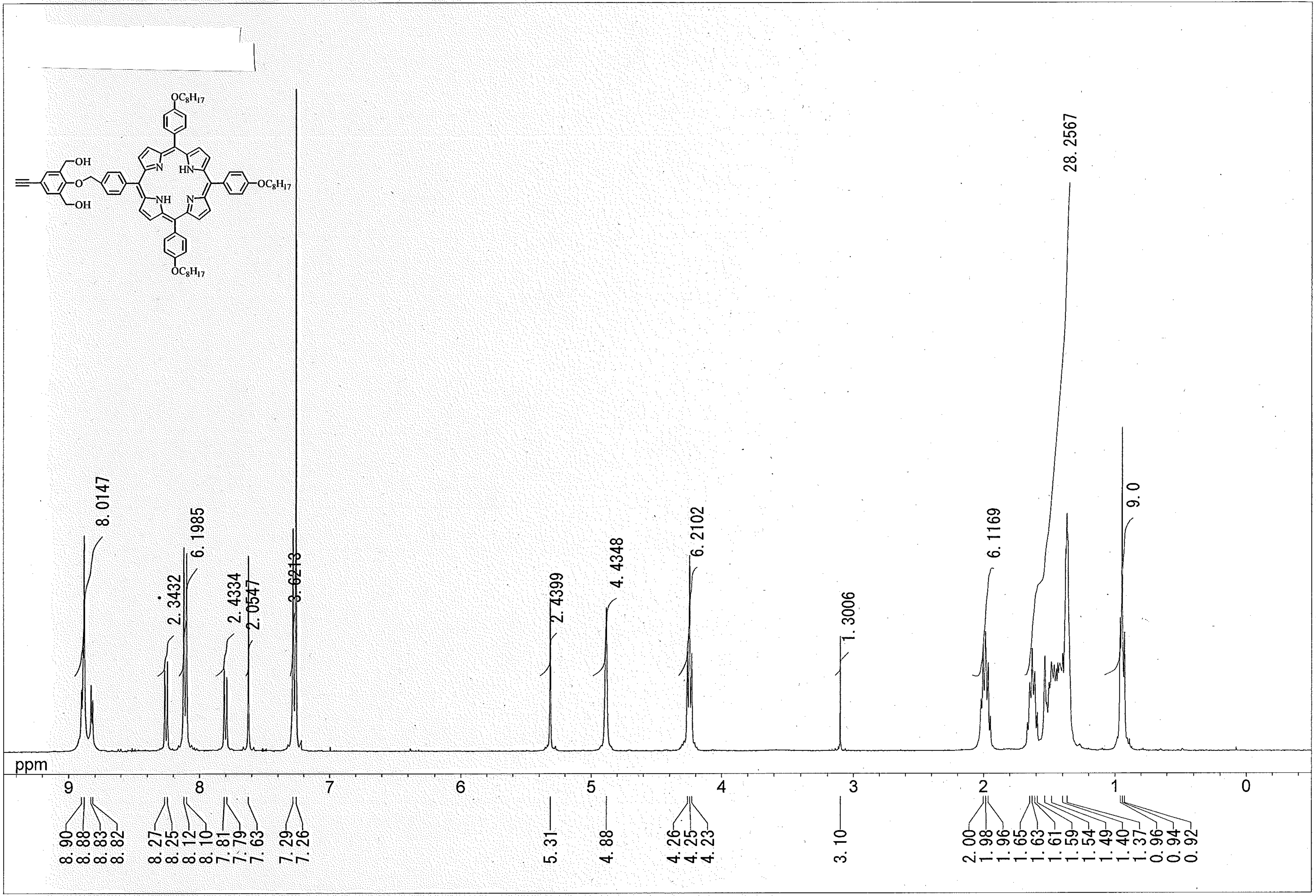

OC8H17
OH
O
N
HN
NH
OC8H17
OH
OC8H17
8.0147
2.3432
6.1985
2.4334
2.0547
3.6213
2.4399
4.4348
6.2102
1.3006
6.1169
28.2567
9.0
ppm
9
8
7
6
5
4
3
2
1
0
8.90
8.88
8.83
8.82
8.27
8.25
8.12
8.10
7.81
7.79
7.63
7.29
7.26
5.31
4.88
4.26
4.25
4.23
3.10
2.00
1.98
1.96
1.65
1.63
1.61
1.59
1.54
1.49
1.40
1.37
0.96
0.94
0.92

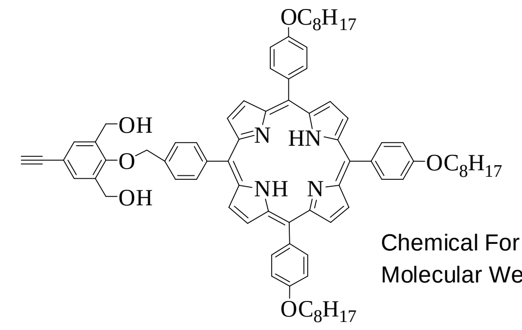

Chemical Formula: $C_{79}H_{88}N_4O_6$
Molecular Weight: 1189.57

monomer **1**

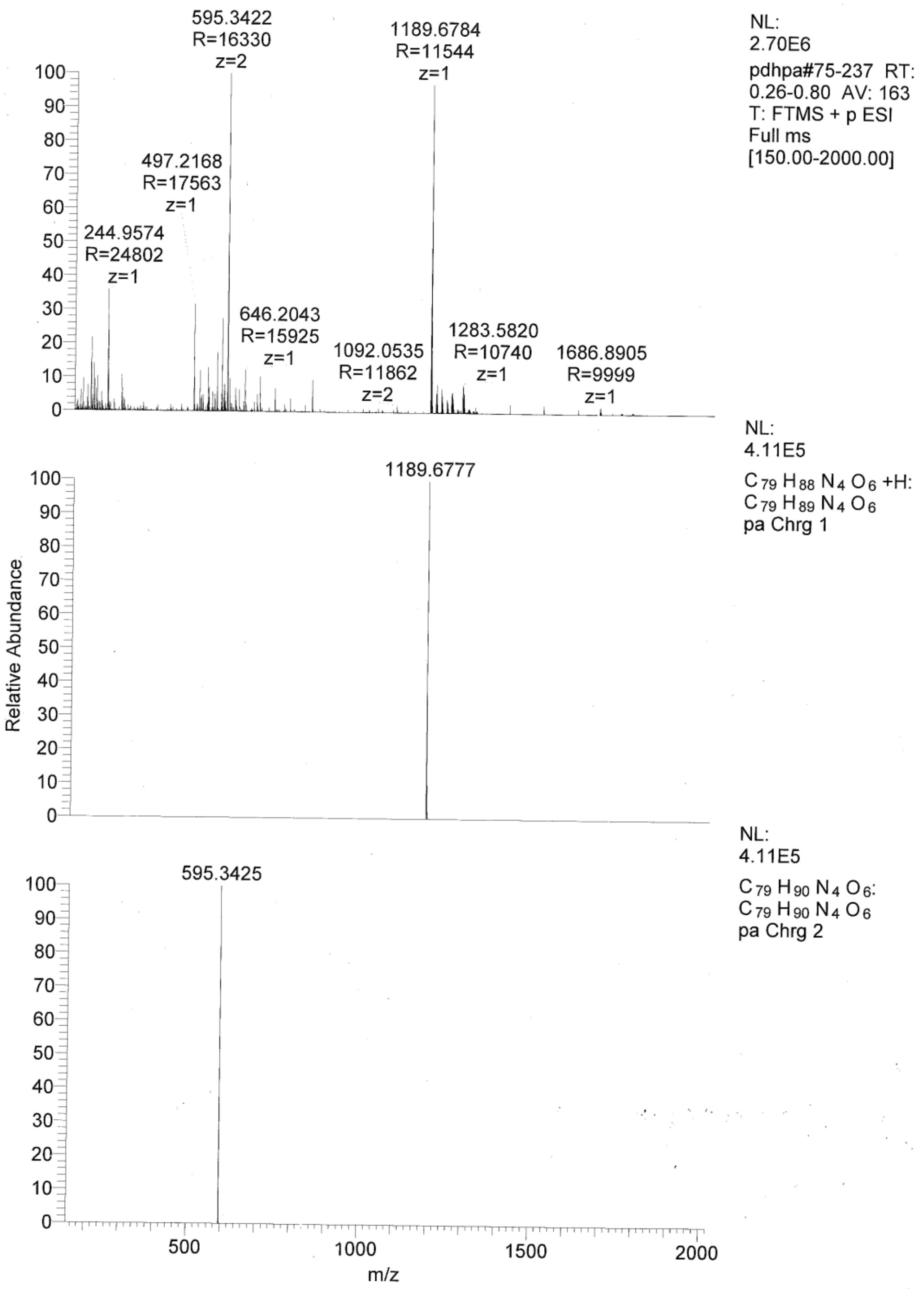

Mass spectrum of monomer **1**
